# Ustekinumab trough concentration affects clinical and endoscopic outcomes in patients with refractory Crohn’s disease: a Chinese real-world study

**DOI:** 10.1186/s12876-021-01946-8

**Published:** 2021-10-18

**Authors:** Jia-yin Yao, Min Zhang, Wei Wang, Xiang Peng, Jun-zhang Zhao, Tao Liu, Zhi-wei Li, Hai-tian Sun, Pinjin Hu, Min Zhi

**Affiliations:** 1 Department of Gastroenterology, Guangdong Provincial Key Laboratory of Colorectal and Pelvic Floor Disease, The Sixth Affiliated Hospital of Sun Yat-Sen University, 26th Yuancun the Second Road, Guangzhou, 510655 Guangdong Province China; 2 Department of Medical Statistics, University of Sun Yat-Sen University, Guangzhou, Guangdong Province China; 3 Special Inspection Project Department, Guangzhou Huayin Medical Examination Center, Guangzhou, Guangdong Province China

**Keywords:** Crohn’s disease, Ustekinumab, Trough concentration, Clinical remission, Endoscopic remission, Therapeutic drug monitoring

## Abstract

**Background:**

Ustekinumab (UST), a newly-used biologic targeting p40 subunit of IL12 and IL23 in China, exerts a confirmed therapeutic effect on the induction and maintenance therapies for refractory Crohn’s disease (CD). Therapeutic drug monitoring based on trough and antibody concentration is of core importance when treating patients who lose response to UST. We aimed to analyze the UST exposure–response relationship in CD treatment in the real-world setting.

**Methods:**

We retrospectively enrolled patients with CD who received UST between March 1, 2020 and May 31, 2021, at the inflammatory bowel disease (IBD) center of the Sun Yat-Sun Affiliated Sixth Hospital. Baseline characteristic information, biomarker examination, clinical outcomes determined by the Crohn’s disease activity index (CDAI), and endoscopic outcomes evaluated using a simple endoscopic score for Crohn’s disease (SES-CD) at week 16/20 were collected. The optimal UST cut-off trough concentration was identified using receiver operating characteristic curve (ROC) analysis.

**Results:**

Nineteen eligible patients were included in the study, the mean age was 29.1 ± 9.1 years and the mean disease duration was 5.5 ± 4.7 years. At the initiation of the study, 89.5% of the patients had been exposed to prior biologics, 42.1% had previous CD-related surgeries, and 52.6% had perianal diseases. At week 16/20 after the UST initiation, clinical response, clinical remission, endoscopic response, and endoscopic remission were 89.5%, 84.2%, 42.2%, and 73.7%, respectively. The cut-off optimal trough concentration for UST was 1.12 μg/mL, as determined by the ROC with an area under the curve (AUC) of 0.78, sensitivity of 87.5%, and specificity of 72.7%. Patients with a UST trough concentration > 1.12 μg/mL had a significantly higher rate of endoscopic remission than those without (70.0% vs. 11.1%, *P* = 0.02).

**Conclusions:**

UST is an effective therapeutic option for refractory CD treatment. A UST trough concentration above 1.12 μg/mL was associated with endoscopic remission at week 16/20 after UST initiation.

*Trial registration* This study was approved and retrospectively registered by the Ethics Committee of Sun Yat-Sen University (2021ZSLYEC-066, March 29, 2021) and the Clinical Trial Registry (NCT04923100, June 10, 2021).

**Supplementary Information:**

The online version contains supplementary material available at 10.1186/s12876-021-01946-8.

## Background

With the evolved “treat-to-target” approach [1], the ultimate aim of Crohn’s disease (CD) treatment has moved from clinical remission to mucosal healing (defined as no ulcerations in any bowel segment [SES-CD ranging from 0 to 2)] [2], or even “deep remission” [3]. Patients who achieve mucosal healing with the early use of intensive therapeutic strategies have been reported to have better long-term outcomes, and to some extent, the progress of CD may have been altered [4].

Ustekinumab (UST) is a human IgG1 that targets the shared p40 subunit of interleukin 12 (IL12) and IL23 [5]. According to UNITI trials [6, 7], UST showed high efficacy in both induction and maintenance therapies for moderate-to-severe CD. Clinical data for the use of UST in China as a CD treatment are currently insufficient, as it has only been in clinical use for one year. Our inflammatory bowel disease (IBD) center reported for the first time in China that UST had a high short-term efficacy with clinical and endoscopic remission rates of 88.9% and 28.6% at week 16/20 after UST initiation, respectively [8]. For those with poor response to UST, therapeutic drug monitoring (TDM) is of great importance [9]. It has been reported that serum concentrations of UST are proportional to the dose and are associated with clinical efficacy [7]. A study from Finland revealed that 39% of patients with refractory CD required UST dose optimization by shortening the medication interval time [10]. However, the efficaciousness of the drug trough concentration and the adequate administration route are still debated worldwide [11–13].

In this single-center retrospective study, we aimed to evaluate the relationship between UST trough concentrations and clinical efficacy, including disease activity improvement, biomarker normalization, and endoscopic amelioration. Understanding the exposure–response relationship and identification of the optimal trough concentration threshold may ultimately facilitate UST treatment optimization.

## Methods

### Patients

Consecutive adult patients with a confirmed CD diagnosis and UST administration were enrolled between March 1, 2020 and May 31, 2021, at the IBD center of the Sun Yat-Sun Affiliated Sixth Hospital, Guangdong, China. This study was approved by the Ethics Committee of Sun Yat-Sen University (2021ZSLYEC-066) and the Clinical Trial Registry (NCT04923100). Due to the retrospective study design, which used anonymous data, written informed consent from the patients was waived.

### Study design

A comprehensive diagnosis of CD is made based on clinical symptoms, laboratory examination, imaging, endoscopy, and pathological findings according to internationally accepted criteria [14, 15]. Diseases were classified using the Montreal classification system [16]. In this study, L4 disease location referred specifically to isolated L4, including esophagogastroduodenal, jejunal, and proximal ileal disease. UST was administered intravenously to moderate-to-severe patients at a dose of 6 mg/kg (520 mg, 390 mg, and 260 mg for patients who weighed above 85 kg, between 65 and 85 kg, and below 65 kg, respectively) at week 0, followed by 90 mg subcutaneously every 12 (q12w) or 8 weeks (q8w) for maintenance therapy. Baseline characteristics including age, gender, disease duration, serum C-reactive protein (CRP) level, previous surgery, previous biologic exposure, and concomitant medication were extracted from the electronic medical records. All patients underwent endoscopy prior to UST initiation and at week 16/20 after UST therapy (approximately the third administration of UST). UST trough and antibody concentrations were detected immediately before each UST administration. Patients who lacked endoscopic data or UST trough and antibody concentrations data and those without a confirmed diagnosis of CD or a follow-up period of less than 16 weeks were excluded. The concomitant immunosuppressants included azathioprine (AZA), methotrexate (MTX), cyclophosphamide (CTX), and thalidomide.

### UST trough and antibody concentration measurements

UST drug and antibody concentrations were detected using an enzyme-linked immunosorbent assay (IDKmonitor® Ustekinumab drug level ELISA kit and Ustekinumab free ADA ELISA kit were purchased from Immundiagnostik AG, Germany). Trough and antibody concentrations were measured in all patients at every visit before UST administration (at baseline, week 8, and week 16/20).

### Outcomes and definition

The main outcome was endoscopic remission at week 16/20. The secondary outcomes were clinical remission, steroid-free clinical remission, clinical response, and endoscopic response at week 16/20. Clinical remission was defined as a Crohn’s disease activity index (CDAI) below 150, while clinical response was defined as a reduction of CDAI > 70 [17]. Endoscopic remission was defined as a simple endoscopic score for Crohn’s disease (SES-CD) ≤ 2, while endoscopic response as defined as a reduction of SES-CD > 50% baseline [18, 19]. Two expert IBD endoscopists assessed and calculated the SES-CD scores. A serum CRP level < 3 mg/L was considered as biomarker normalization.

### Statistical analysis

Continuous variables are presented as mean ± standard deviation (SD) or the median and interquartile range (IQR), while categorical variables are presented as percentages or proportions. Between-group comparisons were performed using the chi-square test, Fisher’s exact test, or Student’s t-test as appropriate. The optimal cutoff point for UST trough concentration was evaluated by receiver operating characteristic curve (ROC) and the area under the curve (AUC) with sensitivity and specificity calculated. Statistical significance was defined as a 2-tailed *P* value < 0.05. All analyses were performed using the SPSS software version 22.0. Two experienced statistical experts (Jin-xin Zhang and Zhi-wei Li) from the Department of medical statistics, University of Sun Yat-Sen University helped with the statistical analysis in this study.

## Results

### Patients

**Fig. 1:**
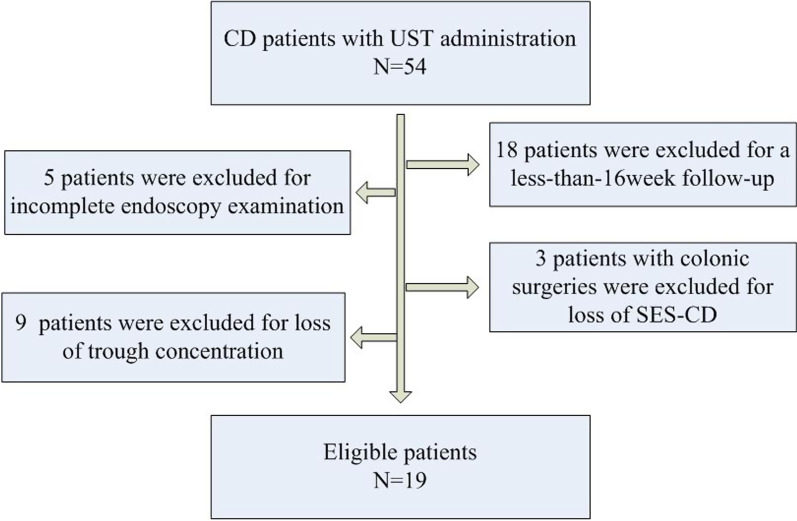
Flowchart of patient recruitment

Fifty-four patients with a confirmed diagnosis of CD and UST administration were enrolled. Eighteen patients were excluded for a less than 16-week follow-up period. Seventeen patients were excluded for incomplete data (five without endoscopy examination, nine without trough concentration at week 16/20, and three without SES-CD for the sake of colonic surgeries) (Fig. 1). Thus, nineteen eligible patients were finally included in this study. The mean age was 29.1 ± 9.1 years, and the mean disease duration was 5.5 ± 4.7 years. At the beginning of the study, 89.5% of patients had been exposed to prior biologics, 42.1% had previous CD-related surgeries, and 52.6% had a perianal disease. The patient baseline characteristics are listed in Table1.

**Table 1:** Patients baseline characteristic information

Index	Cohort (n = 19)
Age, y, mean ± SD	29.1 ± 9.1
Male, n (%)	11 (57.9)
*Montreal classification, n (%)*	
Age at diagnosis	
< 16	0 (0)
16–40	17 (89.5)
> 40	2 (10.5)
Behavior	
B1	1052.6)
B2	5 (26.3)
B3	4 (21.1)
Location	
L1	2 (10.5)
L2	1 (5.3)
L3	16 (84.2)
L4	0 (0)
Disease duration, y, mean ± SD	5.5 ± 4.7
BMI at diagnosis, mean ± SD	19.5 ± 2.4
EIM, n (%)	1 (5.3)
Perianal disease, n (%)	10 (52.6)
Previous biologics exposure, n (%)	17 (89.5)
1	15 (78.9)
2	1 (5.3)
3	0 (0)
4	1 (5.3)
Intestinal surgeries, n (%)	8 (42.1)
Concomitant drugs, n (%)	
Corticosteroid	1 (5.3)
Thiopurine	1 (5.3)
UST administration interval, n (%)	
Q12w	14 (73.7)
Q8w	5 (26.3)

### Clinical and endoscopic outcomes

**Fig. 2:**
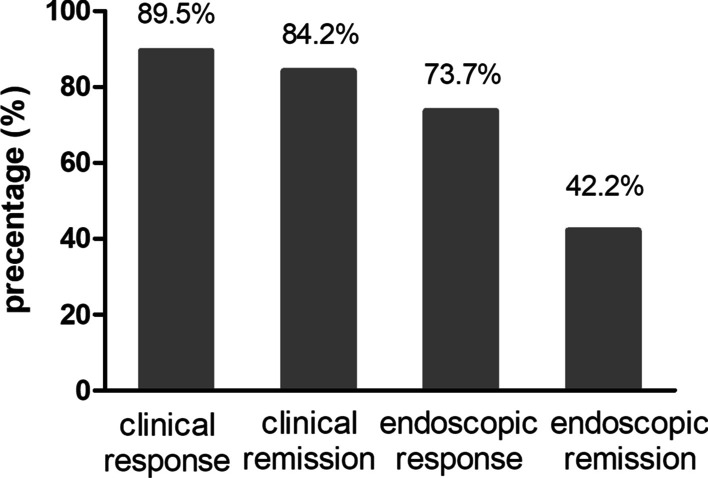
Clinical and endoscopic outcomes at week 16/20 after UST initiation

After UST administration, mean CDAI scores significantly dropped from 220.5 ± 58.8 to 92.4 ± 48.5 (n = 19, *P* < 0.001), while SES-CD scores decreased from 11.2 ± 6.1 to 4.4 ± 4.2 (n = 19, *P* < 0.001) at week 16/20. The proportion of patients with normal CRP levels tended to increase from 5.3% at the baseline to 42.1% at week 16/20 (n = 19, *P* = 0.474). At week 16/20 after UST initiation, clinical response, clinical remission, endoscopic response, and endoscopic remission were 89.5%, 84.2%, 42.2%, and 73.7%, respectively (Fig. 2). Furthermore, the B2 phenotype was associated with a lower clinical response rate (*P* = 0.044) (Additional file 1: Figure S1b). Disease location (Additional file 1: Figure S1a) and UST administration interval (Additional file 3: Table S1) did not alter the outcomes.

### UST trough and antibody concentrations

**Fig. 3:**
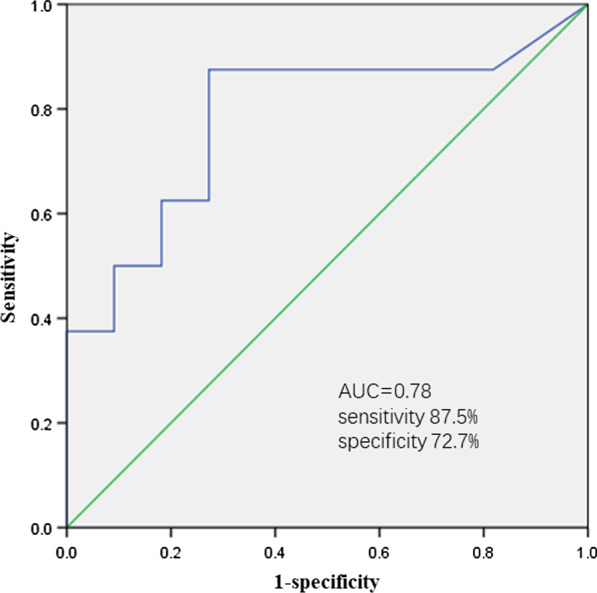
Receiver-operating curve analysis for endoscopic remission based on UST trough concentration with optimal UST trough level cut-off of 1.12 μg/mL

**Fig. 4:**
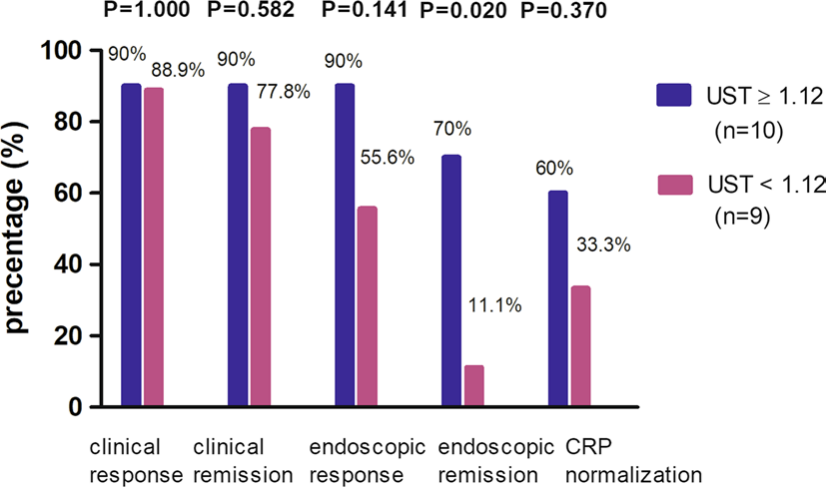
Clinical and endoscopic outcomes based on UST trough concentrations

Mean UST trough concentration was 2.04 ± 0.64 μg/mL at week 16/20. We determined the cut-off optimal trough concentration of 1.12 μg/mL by predicting endoscopic remission using ROC with an AUC of 0.78, sensitivity of 87.5%, and specificity of 72.7% (Fig. 3). Patients with a UST trough concentration > 1.12 μg/mL had a significantly higher rate of endoscopic remission than those without (70.0% vs. 11.1%, *P* = 0.02). Clinical response (90.0% vs. 88.9%, *P* = 1.000), clinical remission (90.0% vs. 77.8%, *P* = 0.582), endoscopic response (90.0% vs. 55.6%, *P* = 0.141), and CRP normalization (60.0% vs. 33.3%, *P* = 0.370) rates for patients with trough levels > 1.12 μg/mL tended to be higher than those without (Fig. 4). However, a quartile analysis of UST trough concentration demonstrated that there was no significant dose response for clinical response (*P* = 0.534), clinical remission (*P* = 0.783), endoscopic response (*P* = 0.068), or endoscopic remission (*P* = 0.061) (Additional file 2: Figure S2). In this study, none of the patients had detectable UST antibody concentrations.

## Discussion

This study demonstrated an optimal cut-off UST trough concentration of 1.12 μg/mL and an exposure–response relationship between the UST trough level and endoscopic remission, implying that maintaining sufficient trough concentrations is closely related to positive treatment outcomes.

Launching an adequate therapeutic strategy for refractory CD is challenging. A vast majority of patients with refractory CD have already lost response to more than one type of biologics or become intolerant to steroids and other immunosuppressants. In China, UST was first approved for the CD treatment in 2020, almost four years after America and Canada. Undoubtedly, UST brings hope for patients with refractory CD in China, regardless of its extremely high cost. Therefore, when patients lose response to UST, therapeutic drug monitoring is of great importance to ensure that UST treatment can be optimized before switching to other therapeutic strategies.

Evidence from novel UNITI-1, UNITI-2, and IM-UNITI trials demonstrated a positive relationship between trough levels and clinical efficacy at 1-year maintenance therapy assessment [6, 7]. A post-hoc analysis of the IM-UNITI cohort revealed that UST trough concentrations > 0.8 μg/mL were the optimal cut-off point with an AUC of 0.64. In a real-world setting, a prospective study conducted by Battat et al. demonstrated that a UST trough level > 4.5 μg/mL at week 26 or beyond was associated with biomarker normalization and clinical remission [20]. Another prospective open-label cohort study showed that week 16 UST concentrations ≥ 2.3 μg/mL and week 24 concentrations ≥ 1.9 μg/mL were associated with endoscopic response at week 24 [21]. In our study, we identified an AUC of 0.78, for endoscopic remission and UST concentration, with an optimal cut-off of 1.12 μg/mL, verifying the relationship between UST pharmacokinetics and exposure–response, which is in accordance with previous studies. The UST concentration cut-off point was lower than those previously reported, which is partly attributed to the different detection methods. To the best of our knowledge, this is the first study on UST pharmacokinetics and the exposure-outcome relationship reported in China.

Furthermore, the immunogenicity of UST was much lower than that of the anti-TNF agents. According to the UNITI series trials, the incidence of antibodies against UST was 0.2% after induction and 2.3% one year after UST initiation [7]. No antibodies against UST were observed in the present study. Only one patient in our study had concomitant thiopurine at the initiation of UST, and in-depth evaluations of the efficacy of the combined therapies were not feasible. It was reported that the containment of immunosuppressants did not affect UST trough concentration nor immunogenicity, which suggested that combined therapy with an immunosuppressant was not necessary [22]. This was confirmed by Hu et al. [23], who recently conducted a retrospective study including 549 patients with IBD. They concluded that there was no difference in clinical response or remission with combined therapy compared with monotherapy at week 14, 30, or 54. Moreover, a meta-analysis by Yzet et al. [24] supported the same view. In contrast, studies conducted by Ma et al. [25, 26] and Wils et al. [27] suggested that combined therapy might improve the clinical efficacy of UST. Large randomized controlled trials are thus required to provide further evidence.

Certain factors may influence the UST trough levels. For example, patients who showed a negative response to previous anti-TNF agents, those with wide-range intestinal lesions, deep ulceration, severe malnutrition, and high CRP levels might contribute to the low trough concentration and poor response to the UST treatment [21, 28]. Therefore, identifying patients with risk factors in advance helps launch early-onset therapeutic drug monitoring to achieve better trough levels and clinical responses.

The UST intensification strategies are discussed and unstandardized. Dose escalation by shortening administration intervals from q12w to q8w or even q4w may decrease the Harvey-Bradshaw index in patients with refractory CD [29]. Re-induction with intravenous UST might be an optimization option for patients with inadequate response at the q4w medication interval. A multicenter retrospective cohort study involving 142 patients from 22 centers and 14 countries demonstrated that 51% and 39% of patients achieved clinical response and remission, respectively, after dose escalation to q4w or q6w, with or without a combination of intravenous re-induction [30]. Thus, intensive treatment ensures that patients with refractory CD respond better to UST treatment. In this retrospective study, the mean UST trough concentration tended to be higher at the q8w than the q12w UST interval, although the difference was not statistically significant. Nevertheless, no significant differences in outcomes could be found in patients with different UST intervals, which could be attributed to the small sample size. Therefore, prospective studies with large sample sizes are required to standardize UST intensification strategies.

### Strengths and limits of the study

Our IBD center is one of the largest in China; therefore, complete clinical and endoscopic data, standardized assessment, and real-life setting were the strengths of this study. However, there were also some limitations. First, the retrospective study design provided relatively low-grade evidence. Second, the limited sample size might have led to underpower analysis. Finally, we only focused on the outcomes at week 16/20 and thus lacked long-term evaluation data.

## Conclusions

In conclusion, UST is effective in treating refractory CD with confirmed efficacy both in induction and maintenance therapies. In this real-world study, we identified an ideal UST trough concentration cut-off point of 1.12 μg/mL, and patients with trough levels > 1.12 μg/mL are reported to be associated with higher endoscopic remission rates. However, more multicenter randomized controlled trials with larger sample sizes and longer follow-up periods are warranted to provide stronger evidence for individual therapies for refractory CD.

## Supplementary Information

**Additional file 1**. Clinical and endoscopic outcomes based on location (a, left) and disease behavior (b, right).

**Additional file 2**. A quartile analysis of UST trough concentration did not demonstrate a dose response for clinical and endoscopic outcomes.

**Additional file 3**. Clinical and endoscopic outcomes in patients with different Ustekinumab administration interval.

## Data Availability

All data generated or analyzed during this study are included in this published article [and its additional files].

## Abbreviations

CD: Crohn’s disease
IBD: Inflammatory bowel disease
CDAI: Crohn’s disease activity index
SES-CD: Simple endoscopic score for Crohn’s disease
UST: Ustekinumab
AUC: Area under the curve
ROC: Receiver operating characteristic curve
TDM: Therapeutic drug monitoring
CRP: C-reactive protein
AZA: Azathioprine
MTX: Methotrexate
CTX: Cyclophosphamide
