# Supplementary figures and images for: Ustekinumab trough concentration affects clinical and endoscopic outcomes in patients with refractory Crohn’s disease: a Chinese real-world study
Source: BMC Gastroenterol. 2021 Oct 18;21:380. doi: 10.1186/s12876-021-01946-8 (PMC8522105; doi:10.1186/s12876-021-01946-8)

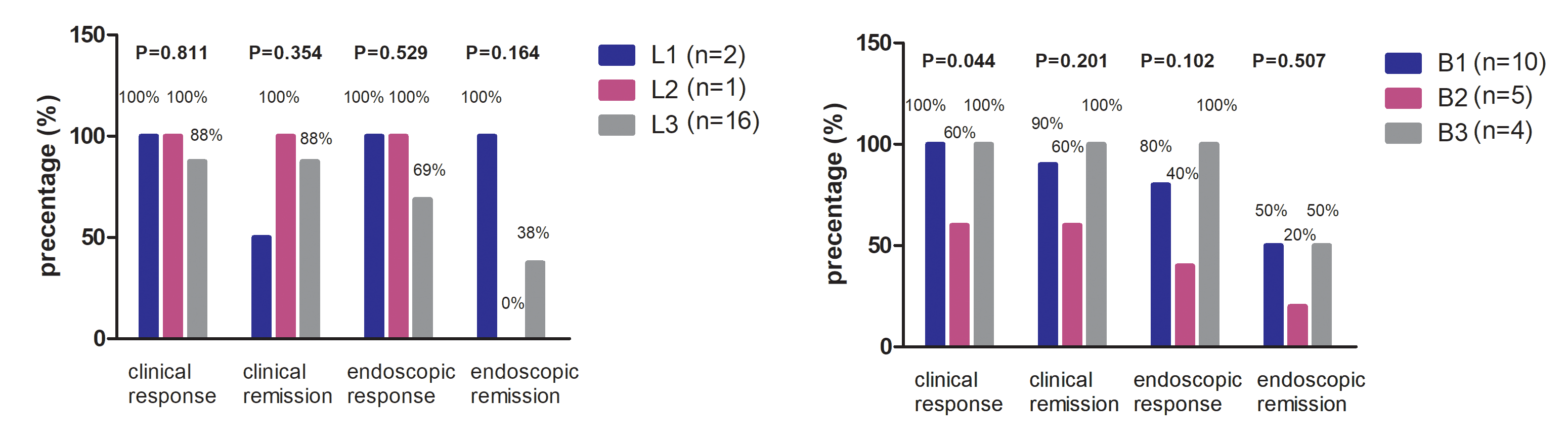

Supplement: Supplementary file 1 — Additional file 1. Clinical and endoscopic outcomes based on location (a, left) and disease behavior (b, right). [file 12876_2021_1946_MOESM1_ESM.tif]

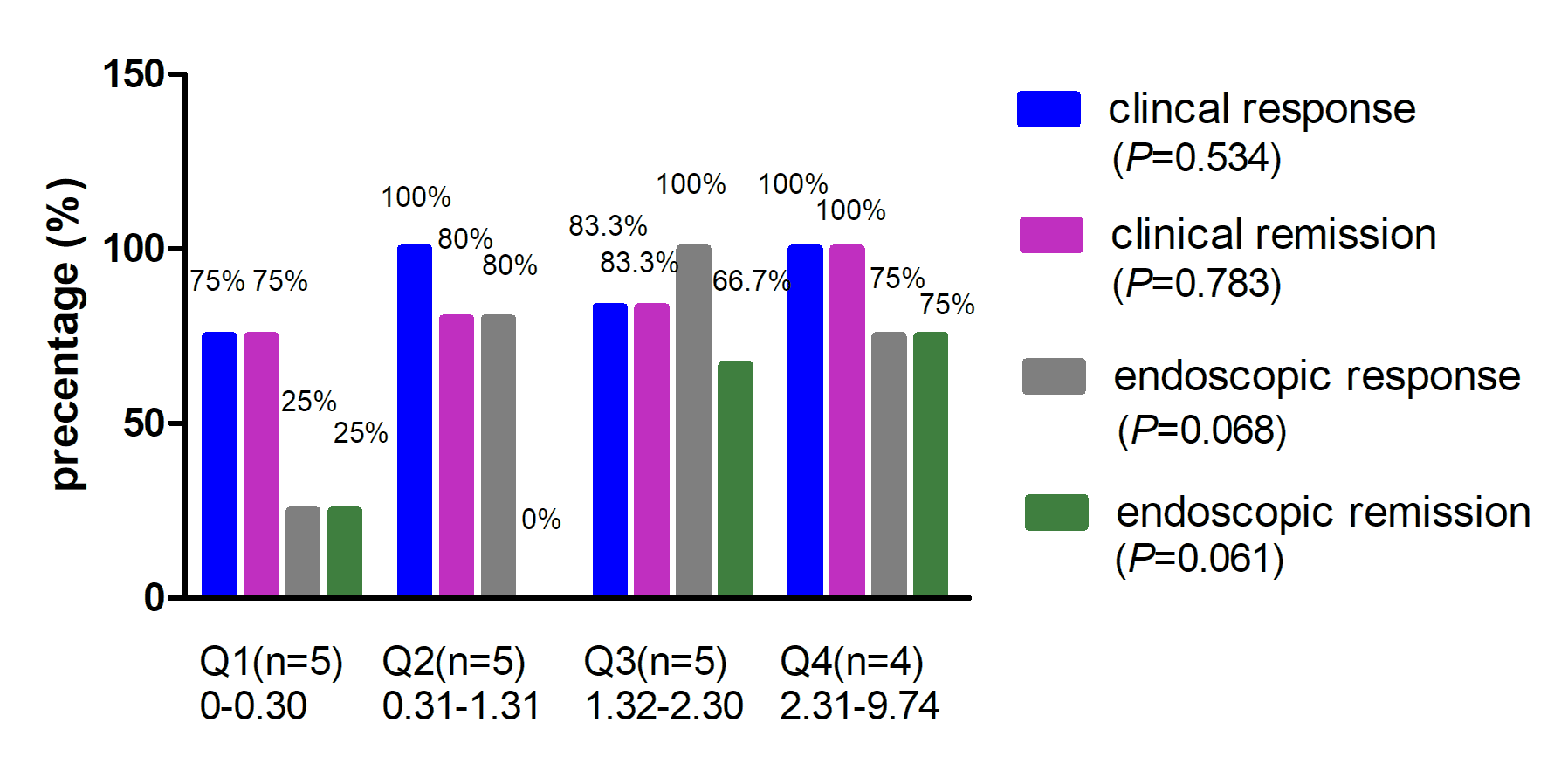

Supplement: Supplementary file 2 — Additional file 2. A quartile analysis of UST trough concentration did not demonstrate a dose response for clinical and endoscopic outcomes. [file 12876_2021_1946_MOESM2_ESM.tif]
