# Supplementary material for: Ustekinumab trough concentration affects clinical and endoscopic outcomes in patients with refractory Crohn’s disease: a Chinese real-world study
Source: BMC Gastroenterol. 2021 Oct 18;21:380. doi: 10.1186/s12876-021-01946-8 (PMC8522105; doi:10.1186/s12876-021-01946-8)
Supplement: Supplementary file 3 — Additional file 3. Clinical and endoscopic outcomes in patients with different Ustekinumab administration interval. [file 12876_2021_1946_MOESM3_ESM.docx]

Supplementary Table 1 Clinical and endoscopic outcomes in patients with different Ustekinumab administration interval.

|  | Every 8 weeks (n=5) | Every 12 weeks (n=14) | P value |
| --- | --- | --- | --- |
| Mean UST concentration, μg/mL, mean$\pm$SD | 3.59$\pm$1.62 | 1.49$\pm$0.62 | 0.051 |
| Clinical response (%) | 80 | 93 | 0.468 |
| Clinical remission (%) | 100 | 79 | 0.530 |
| Endoscopic response (%) | 100 | 64 | 0.257 |
| Endoscopic remission (%) | 60 | 36 | 0.603 |
